# Adaptation of *Pseudomonas aeruginosa* to Phage PaP1 Predation via *O*-Antigen Polymerase Mutation

**DOI:** 10.3389/fmicb.2018.01170

**Published:** 2018-06-01

**Authors:** Gang Li, Mengyu Shen, Yuhui Yang, Shuai Le, Ming Li, Jing Wang, Yan Zhao, Yinling Tan, Fuquan Hu, Shuguang Lu

**Affiliations:** Department of Microbiology, Army Medical University, Chongqing, China

**Keywords:** phage therapy, phage resistance, adsorption inhibition, *Pseudomonas aeruginosa*, *O*-antigen polymerase, biofilm

## Abstract

Adaptation of bacteria to phage predation poses a major obstacle for phage therapy. Bacteria adopt multiple mechanisms, such as inhibition of phage adsorption and CRISPR/Cas systems, to resist phage infection. Here, a phage-resistant mutant of *Pseudomonas aeruginosa* strain PA1 under the infection of lytic phage PaP1 was selected for further study. The PaP1-resistant variant, termed PA1RG, showed decreased adsorption to PaP1 and was devoid of long chain *O*-antigen on its cell envelope. Whole genome sequencing and comparative analysis revealed a single nucleotide mutation in the gene PA1S_08510, which encodes the *O*-antigen polymerase Wzy that is involved in lipopolysaccharide (LPS) biosynthesis. PA1_Wzy was classified into the O6 serotype based on sequence homology analysis and adopts a transmembrane topology similar to that seem with *P. aeruginosa* strain PAO1. Complementation of gene *wzy in trans* enabled the mutant PA1RG to produce the normal LPS pattern with long chain *O*-antigen and restored the susceptibility of PA1RG to phage PaP1 infection. While *wzy* mutation did not affect bacterial growth, mutant PA1RG exhibited decreased biofilm production, suggesting a fitness cost of PA1 associated with resistance of phage PaP1 predation. This study uncovered the mechanism responsible for PA1RG resistance to phage PaP1 via *wzy* mutation and revealed the role of phages in regulating bacterial behavior.

## Introduction

Bacteriophages (phages) are viruses that can infect and kill the bacterial hosts specifically. Interaction between phages and their bacterial hosts is important in molecular biology research and has been extensively studied for decades (Chaturongakul and Ounjai, 2014). In various environments, phages and bacteria work toward into an endless state of co-evolutionary competition and equilibrium (De Smet et al., 2017). On the one hand, the high abundance of phages, which outnumber bacteria by approximately 10-fold, makes the encounter of bacteria and phage invaders possible in every ecosystem (Sharma et al., 2017). To survive and/or escape phage predation, bacteria have evolved and acquired sets of resistance mechanisms, including prevention of phage adsorption to cell surfaces and subsequent injection of phage genomes, targeted cleavage of injected nucleic acids via restriction-modification (R-M) system and CRISPR/Cas system, and even suicide of phage-infected cells via abortive-infection (Abi) system (Labrie et al., 2010; Samson et al., 2013). On the other hand, despite the presence of these arsenals, phages have also adapted to overcome bacterial defense systems through several counter-strategies (Samson et al., 2013), such as mutation in specific phage genes (Pepin et al., 2008; Michel et al., 2010), phage genome rearrangement, and exchange with other viral or bacterial sequences (Labrie and Moineau, 2007). The constant competition and co-evolution contribute greatly to the genetic diversity of both bacteria and phages on the biosphere (Chaturongakul and Ounjai, 2014).

*Pseudomonas aeruginosa* is a Gram-negative opportunistic pathogen that causes various infections mainly in immunocompromised individuals, especially for those suffering from burn wounds, cancer and cystic fibrosis (Stover et al., 2000; Turner et al., 2015). The notorious characteristics of metabolic versatility, biofilm formation, and drug resistance make *P. aeruginosa*-related infections very difficult to eradicate in clinical settings (Mathee et al., 2008; Valot et al., 2015). Phages represent a promising alternative to traditional antibiotics for treating bacterial infections (Nobrega et al., 2015), particularly for multidrug-resistant and biofilm infections (Nobrega et al., 2015; Chan et al., 2016a). Despite the impressive specificity and efficiency of phage therapy, phage resistance still poses a major obstacle to its broad application (Nilsson, 2014). Therefore, a comprehensive understanding of the interactions between phages and bacteria, as well as phage resistance mechanisms, is still urgently required to predict and limit potentially undesired outcomes during phage-based applications.

*Pseudomonas aeruginosa* strain PA1 was originally isolated from a patient suffering from a respiratory tract infection and resists treatment by multiple antibiotics (Lu et al., 2015; Li et al., 2016b). Recent work has shown that upon predation by the lytic phage PaP1, PA1 produced phage-resistant variants at a frequency of 3 × 10^-5^, and these variants could be classified into two major phenotypes: one produces red pigment homogentisic acid (Red mutant), whereas the other displays blue–green pigment pyocyanin (Green mutant) (Le et al., 2014). The features of phage resistance and pigment changes could be stably maintained for these mutants, suggesting potential genetic mutations within the PA1 genome. Comparative genomic analysis revealed a 219.6 kb genomic fragment deletion in the Red mutant (termed PA1r), and this deleted DNA fragment containing the key gene *hmgA* and *galU*. Loss of *hmgA* contributes to the accumulation of a red compound, homogentisic acid, whereas *galU* deletion results in hindering of bacterial lipopolysaccharide (LPS) biosynthesis, which functions as the receptor for phage PaP1 adsorption (Le et al., 2013). By contrast, the Green mutant produces the same pigment (pyocyanin) as the parental strain PA1. However, the underlying molecular basis for the phage PaP1 resistance of the Green mutant remains unknown.

In this study, a Green mutant of *P. aeruginosa* PA1 was characterized and termed as PA1RG. Comparative genomic analysis combined with molecular characterization revealed the crucial role of *O*-antigen polymerase mutation in conferring the phage PaP1 resistance to PA1RG. The results of this work enhances our understanding of phage resistance mechanisms of bacteria.

## Materials and Methods

### Bacteria and Growth Conditions

*Pseudomonas aeruginosa* strains PA1, PA1RG, and PA1RG/*wzy* were cultured in LB broth at 37°C unless otherwise specified. When necessary, 100 μg/ml gentamicin was supplemented for PA1RG/*wzy* culture. The value of OD_600_ for both PA1 and PA1RG cultures was measured every 30 min to profile the growth curve. The production of green pigment pyocyanin was photographed at the early-stationary phase.

### Adsorption Assay

Phage PaP1 attachment ability was determined as previously described with minor modifications (Le et al., 2014). Briefly, log-phase culture of bacteria was washed and resuspended with LB broth to 10^8^ cfu/ml. Phage PaP1 was then added to the bacterial suspension at a final concentration of 10^5^ pfu/ml and was incubated at 37°C for 5 min. The samples were filtered, and phage titer in the supernatants was quantified. Adsorption ability was represented as [(total titer – titer in the supernatant)/total titer] × 100%.

### LPS Profiling

Lipopolysaccharide was isolated using the hot water-phenol method as described previously (Yi et al., 2009). Purified LPS was subjected to 12% SDS-PAGE and visualized by silver staining as formerly described (Fomsgaard et al., 1990).

### SMRT Sequencing of PA1RG Genome

Complete genome sequence of PA1RG was determined on the PacBio RSII instrument (Pacific Biosciences, Menlo Park, CA, United States). Libraries of 5-kb were constructed for PA1RG genomic DNA, and four SMRT cells of the libraries were sequenced with 90-min movies. *De novo* assembly using RS_HGAP_Assembly version 2.0 (Chin et al., 2013), revealed a single contig of 6,500,439 bp in length with 320-fold sequence coverage (Li et al., 2016a).

The genome sequences of strain PA1, PA1RG, and PA1r are available at GenBank under the accession number CP004054, CP012679, and CP004055, respectively.

### Bioinformatics Analysis

Sequence homology was searched using BLAST service at the NCBI website^1^. Multiple alignment of Wzy was performed by Clustal W (Larkin et al., 2007), and visualized using ESPript (Robert and Gouet, 2014). Wzy homologs used for phylogenetic analysis were retrieved from literature (Islam and Lam, 2014; Pan et al., 2016). Phylogenetic tree was constructed by MEGA using the neighbor-joining method (Saitou and Nei, 1987; Tamura et al., 2013). The transmembrane topology of Wzy was analyzed by software TMHMM 2.0 (Krogh et al., 2001).

### Construction of the Complementary Strain PA1RG/*wzy*

The intact gene *wzy* as well as its promoter region was amplified from PA1 genome using forward primer 5′-GGGGTACCCTTGCCGTCACTTTCTCCGA-3′ (KpnI site underlined) and reverse primer 5′-AACTGCAGGGAGTTGGCGCATATGCATA-3′ (PstI site underlined). The obtained PCR fragment was cloned into the KpnI/PstI sites of plasmid pUCP24 (West et al., 1994), resulting in pUCP*wzy*. Construct pUCP*wzy* was then electroporated into PA1RG to obtain the complementary strain PA1RG/*wzy* as described previously (Li et al., 2017).

### Spot Assay

Sensitivity of PA1, PA1RG, and PA1RG/*wzy* to phage PaP1 was determined as described previously (Bondy-Denomy et al., 2016). Briefly, bacteria were firstly cultured to log-phase, and 200 μl of aliquot mixed with 3 ml of soft LB agar (0.7%, w/v) was poured onto the LB agar plate (1.5%, w/v) and solidified at room temperature. The phage PaP1 lysate (10^10^ pfu/ml) was serially diluted in 10-fold steps, and 3 μl of dilutions was applied to the lawn of each strain, and bacterial lysis was assessed.

### Pyocyanin Quantitation Assay

Pyocyanin production was quantitated as previously described (Essar et al., 1990). Briefly, a 5 ml of culture supernatant grown in LB broth was extracted with 3 ml of chloroform. After centrifugation, the chloroform layer was re-extracted into 1 ml of 0.2 N HCl to obtain a pink to deep red solution. The absorbance of this solution was quantified at 520 nm.

### Microtiter Dish Biofilm Formation Assay

The microtiter dish biofilm formation assay was performed as previously described (O’Toole, 2011). Briefly, overnight culture of PA1, PA1RG, and PA1RG/*wzy* was diluted 1:100 into fresh LB medium, respectively. Then, 100 μl of aliquot was inoculated into the flat-bottom 96-well dish (Corning, United States) with six replicate wells for each strain. Biofilm was formed at 37°C for 24 h. After incubation, the plate was washed with ddH_2_O to remove unattached cells and media components, followed by staining with 125 μl of 0.1% crystal violet (Sangon Biotech, Shanghai, China). The plate was washed three times with ddH_2_O again and dried at room temperature. 125 μl of 30% acetic acid (Sangon Biotech, Shanghai, China) was added to solubilize the crystal violet, and then transferred to a new flat-bottom microtiter dish. The absorbance was quantified in a plate reader at 550 nm. Three biological repeats were performed for biofilm formation assay.

### Confocal Laser Scanning Microscopy (CLSM)

Biofilm formation examined by CLSM was performed as described previously with minor modifications (Takenaka et al., 2001). Briefly, overnight culture of PA1, PA1RG, and PA1RG/*wzy* was diluted 1:100 into fresh LB medium, respectively. Then, 2 ml of aliquot was inoculated into the glass-bottom cell culture dish (15 mm in diameter, Nest, China), and incubated at 37°C for 24 h. The biofilm was washed with phosphate-buffered saline (PBS), and fixed with 2.5% glutaraldehyde for 1.5 h. After washing with PBS, the biofilm was labeled by 5 μM fluorescent nucleic acid stain SYTO 61 (Invitrogen, United States) at room temperature for 30 min, followed by 50 μg/ml FITC-labeled concanavalin A (FITC-ConA, Sigma-Aldrich, United States) at 37°C for 5 min. The biofilm was visualized using the confocal laser scanning microscope LSM800 (Zeiss, Germany) with 561 nm excitation and 640 nm emission wavelengths for SYTO 61, and 488 and 537 nm for FITC-ConA, respectively.

### Statistical Analysis

Data were processed using GraphPad Prism (GraphPad Software Inc., San Diego, CA, United States). When necessary, Student’s *t*-test was applied, and the difference was considered statistically significant at *p* < 0.05.

## Results

### PaP1 Infection of *P. aeruginosa* Resulted in Phage-Resistant Mutant PA1RG

**FIGURE 1:**
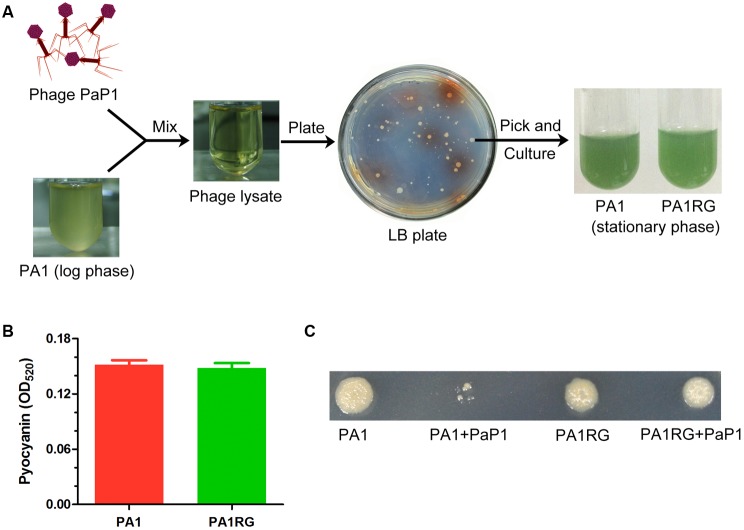
Isolation of phage-resistant mutant PA1RG. **(A)** Diagram of PA1RG separation process. **(B)** Quantitation of pyocyanin production. Data were expressed as mean ± standard deviation (SD) from three biological replicates. **(C)** Dot assay on agar plate showed that PA1RG was steady resistant to phage PaP1.

When the early log-phase culture of *P. aeruginosa* strain PA1 (10^8^ cfu) was infected with phage PaP1 (10^10^ pfu) at an MOI of 100, the cloudy bacterial fluid became transparent, seemingly all the bacteria were killed by phage PaP1 (**Figure 1A**). However, several surviving bacteria grew colonies when plating the transparent lysate on an LB agar plate. This finding suggested that the phage-resistant mutants were selected by PaP1. Approximately two-thirds of the surviving bacteria grew colonies with no pigment produced on the screening plate, and one of these colonies was randomly selected for future analysis. Actually, the selected phage-resistant mutant could produce blue-green pigment in liquid media, which is identical to that of wild-type PA1 (**Figure 1A**). Thus it was named as PA1RG (Resistant Green mutant). No significant difference was identified for pyocyanin production between PA1 and PA1RG (**Figure 1B**), indicating that the biosynthesis of pigment pyocyanin was not impaired in mutant PA1RG. Phage PaP1 could efficiently lyse PA1 but not PA1RG even at high phage titer, confirming the phage-resistant phenotype of PA1RG (**Figure 1C**).

### Whole Genome Single-Molecule Real-Time (SMRT) Sequencing of PA1RG

**FIGURE 2:**
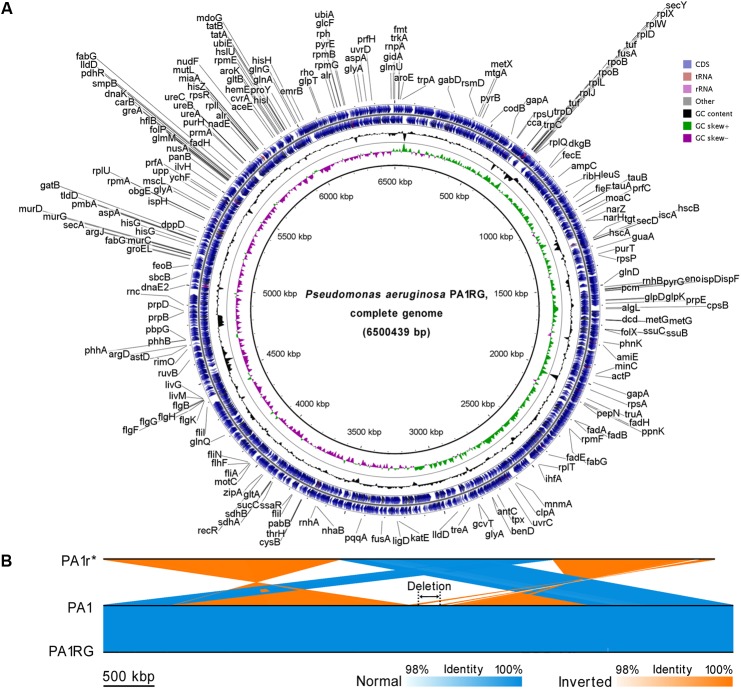
Circular presentation and pairwise comparison of the PA1RG genome. **(A)** Diagram of the *P. aeruginosa* PA1RG genome. The names of 218 annotated genes (also shown in the GenBank file of the PA1RG genome) were indicated in black in the outermost region. The outermost ring depicts the genes on the plus strand, followed by rings depicting the genes on the minus strand, the GC content (black), and GC skew (green/purple). **(B)** Pairwise nucleotide sequence comparison of PA1r, PA1, and PA1RG. The length of the deletion region is 219.6 kb. ^∗^In the GenBank file, the name of the Red mutant is PA1R, which is identical to PA1r.

To probe the possible genetic or epigenetic variations within the PA1RG genome that conferred the phage-resistant phenotype, the PA1RG genomic DNA was extracted and processed by SMRT sequencing, which revealed a single contig of 6,500,439 bp in length with 320-fold sequence coverage (GenBank accession number: CP012679) (Li et al., 2016a). The result of SMRT sequencing revealed that the methylation status of PA1RG did not differ from that of PA1 (data unpublished). The circular genome map of PA1RG is shown in **Figure 2A**. Compared with the genome sequence of PA1, the PA1r (Red mutant) genome contains several large variations, such as inverted regions, contig rearrangements, and a deletion with 219.6 kb in length (**Figure 2B**). By contrast, the PA1RG genome sequence exhibited no large variation compared to that of PA1 (**Figure 2B**).

### Genomic Variation Analyses Revealed the Mutation of Gene PA1S_08510

**FIGURE 3:**
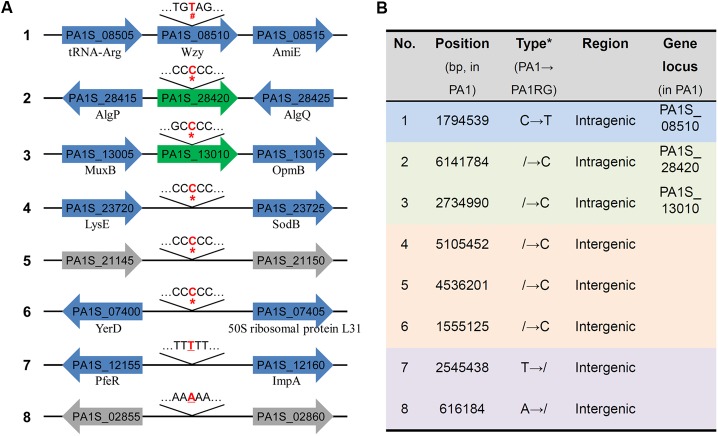
Genetic variations identified within the PA1RG genome. **(A)** Diagram of mutations of the PA1RG genome sequence. Mutated nucleotides were labeled as red and bold letters, the symbol of ^#^, ^∗^, and underline indicates nucleotide substitution, insertion, and deletion, respectively. Green arrows represent pseudo genes, gray arrows represent genes encoding hypothetical protein, blue arrows represent functional genes and products are labeled underneath. Genes were not drawn to the scale. **(B)** Summary of genetic variations within the PA1RG genome. ^∗^The symbol of/indicates absence of nucleotide in corresponding genomic site.

Though no large variation was found in the mutant PA1RG genome, eight single nucleotide mutations were identified compared to the published PA1 genome sequence, including five insertions, two deletions, and one base transition (**Figure 3A**). Further analysis of the local sequence context of these mutated sites indicated that for the seven InDel (insertion/deletion) variations, each of the altered nucleotides was located within a continuous repeat of the same base. Taking number 2 mutation for example, the corresponding DNA sequence 5′-TCCCCCCG-3′ within PA1 was changed to 5′-TCCCCCCCG-3′ after the insertion of the nucleotide cytosine within PA1RG genome. Among the eight variations, five were located in intergenic regions with no regulatory functions predicted, and three were in intragenic regions that matched the PA1 genes PA1S_13010, PA1S_28420, and PA1S_08510, respectively (**Figure 3A**). The detailed genetic variations identified in the PA1RG genome are summarized in **Figure 3B**. Both genes PA1S_13010 and PA1S_28420 were annotated as pseudo genes within the PA1 genome. Gene PA1S_08510 encodes a protein which is 417 amino acids in length, and the transition from cytosine to thymine in gene PA1S_08510 results in a stop codon and premature formation of a truncated product (198 amino acids in length). Thus, we focused our further study on the gene PA1S_08510.

### Gene PA1S_08510 Encodes a Protein Homologous to Wzy, an *O*-Antigen Polymerase

BlastP alignment revealed that protein PA1S_08510 was highly conserved (>90% identity) among various *P. aeruginosa* strains, and almost all of the homologs were recorded as Wzy, an *O*-antigen polymerase encoded by the gene *wzy*. The interval spanning of 25–396 amino acid of PA1S_08510 constituted a conserved domain designated as glyco_rpt_poly (TIGR04370), which is suggested to participate in LPS *O*-antigen biosynthesis (Marchler-Bauer et al., 2017). Therefore, we proposed that PA1S_08510 could be named as PA1_Wzy.

**FIGURE 4:**
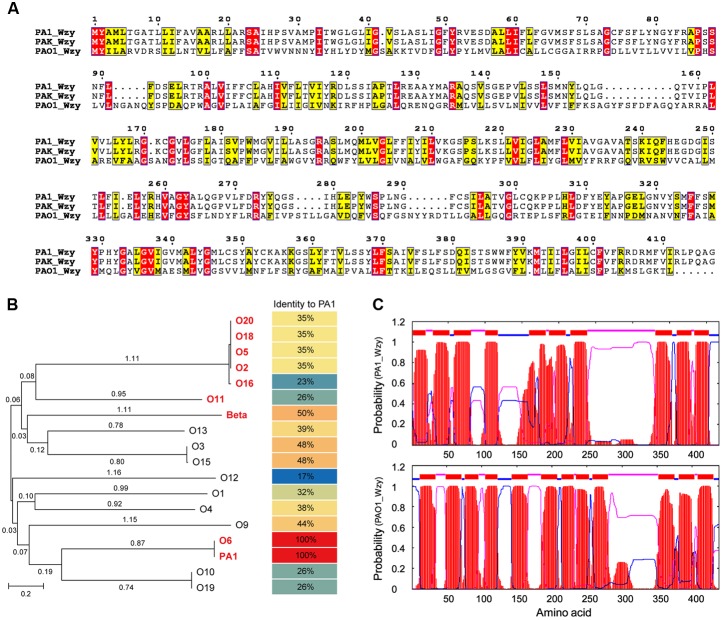
*In silico* analyses of PA1_Wzy. **(A)** Sequence alignment of Wzy. Identical residues are shown as white letters boxed in red, similar residues are written with black characters boxed in yellow. **(B)** Phylogenetic analysis. Wzy homologs belonging to different *P. aeruginosa* serotypes were subjected to phylogenetic analysis. The serotypes labeled in red and bold letters indicate that Wzy activity has been experimentally determined in these serotypes. Beta represents phage D3-coding Wzy that mediates β-linkage of *O*-antigen repeat unit. **(C)** Transmembrane topology analysis. Red: transmembrane helices. Blue: cytoplasmic loops. Pink: periplasmic loops.

PA1_Wzy exhibited 100% identity with Wzy of *P. aeruginosa* strain PAK (serotype O6) (**Figure 4A**), and the functionality of PAK_Wzy (Y880_RS05480) has been genetically confirmed (Pan et al., 2016). By contrast, PA1_Wzy displayed limited sequence identity (35%) with the active Wzy (PA3154) encoded by *P. aeruginosa* strain PAO1 (serotype O5) (Islam et al., 2010) (**Figure 4A**). Phylogenetic and alignment analysis showed that the primary sequences of *O*-antigen polymerase Wzy differ greatly among the *P. aeruginosa* strains that belong to different serotypes (Islam and Lam, 2014) (**Figure 4B**), and PA1_Wzy was classified into the O6 serotype based on sequence homology (**Figure 4B**). Despite the limited sequence conservation between PA1_Wzy and PAO1_Wzy, the two proteins adopt a similar transmembrane topology, especially for the presence of a large periplasmic loop at the C-terminal region (**Figure 4C**).

### *wzy* Mutation Resulted in an LPS Defect in PA1RG

Lipopolysaccharide is a complex and integral component of the bacterial cell envelope and typically consists of three structural domains: lipid A, core oligosaccharide, and the distal *O*-antigen (Lam et al., 2011). In the Wzy-dependent LPS biosynthetic pathway, the *O*-antigen repeat unit is firstly polymerized via Wzy to form the long-chain polymer, followed by anchoring to the lipid core by the ligase, WaaL (Rocchetta et al., 1999). Bacteria lacking Wzy would produce known semi-rough LPS containing lipid A-core plus only one repeat unit on the cell surface (Lam et al., 2011).

**FIGURE 5:**
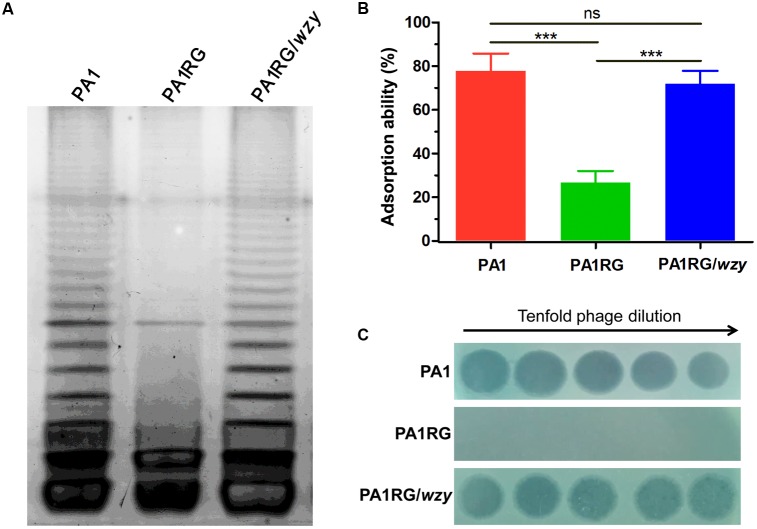
LPS profiling, adsorption assay, and spot assay of PA1RG in comparison with PA1 and PA1RG/*wzy*. **(A)** LPS profiling. Mutant PA1RG produced LPS lacking long chain *O*-antigen. Complementary strain PA1RG/*wzy* displayed the same LPS pattern to that of wild-type PA1. **(B)** Adsorption assay. Decreased PaP1 attachment was observed for mutant PA1RG, which could be complemented by providing *wzy in trans*. Data were expressed as mean ± SD from three biological replicates. ns, not significant. ^∗∗∗^*p* < 0.001. **(C)** Spot assay. Tenfold serial dilutions of PaP1 lysates were applied to the lawns of indicated strains. Phage-resistant PA1RG restored PaP1 susceptibility when bearing the intact gene *wzy*.

To profile the LPS phenotype, the LPS of strain PA1 and PA1RG was extracted and subjected to SDS-PAGE analysis. Silver-stained SDS-PAGE gel showed that wild-type PA1 produced the normal LPS pattern, whereas mutant PA1RG contained the core oligosaccharide capped with one repeat unit, but failed to form the *O*-antigen with high molecular weight (**Figure 5A**), demonstrating the semi-rough LPS phenotype of mutant PA1RG. To further validate the activity of PA1_Wzy, the wild-type *wzy* gene was transformed into mutant PA1RG. The LPS profile of the complementary strain PA1RG/*wzy* was then determined and displayed the same pattern to that of wild-type PA1 (**Figure 5A**). This finding revealed that PA1_Wzy provided *in trans* restored the long chain *O*-antigen biosynthesis in mutant PA1RG and functioned as an active *O*-antigen polymerase.

### Decreased Adsorption Confers PA1RG Resistance to Phage PaP1

The initial and essential step of phage infection is the attachment of phage particles to the bacterial cell surface via receptor-ligand interactions. Adsorption inhibition caused by receptor mutation is usually identified in phage-resistant variants (Labrie et al., 2010). A previous study suggested that phage PaP1 recognizes the long chain *O*-antigen of PA1 LPS as its binding receptor (Le et al., 2013). To determine whether or not the *wzy*-mutation-caused LPS defect could inhibit the adsorption of PaP1 to PA1RG, an adsorption assay was performed. Compared with the ability of the attachment to the wild-type strain PA1, that of phage PaP1 attachment was significantly decreased to mutant PA1RG, but restored to the complementary strain PA1RG/*wzy* (**Figure 5B**). These results confirmed that *wzy*-mutation-caused LPS truncation reduced the adsorption of phage PaP1 to PA1RG.

To further define the role of PA1_Wzy in regulating PA1RG resistance to phage PaP1, a spot assay was performed for the complementary strain PA1RG/*wzy*. Mutant PA1RG restored the susceptibility to phage PaP1 when possessing the intact gene *wzy in trans* (**Figure 5C**), demonstrating that *wzy* mutation caused PA1RG resistance to PaP1 predation.

### Reduced Biofilm Formation for Phage-Resistant PA1RG

**FIGURE 6:**
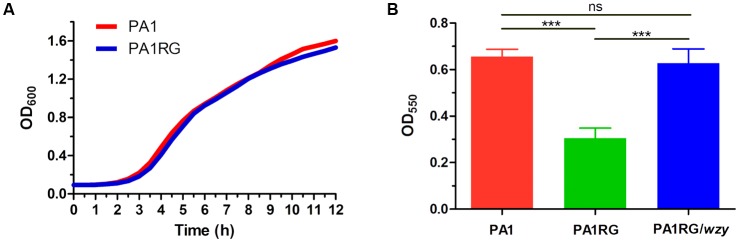
Growth and biofilm assay. **(A)** Growth curves of PA1 and PA1RG that monitored every 30 min. Data represent the average of three biological replicates. **(B)** Microtiter dish biofilm formation assay. Data were expressed as mean ± SD from three biological replicates. ns, not significant. ^∗∗∗^*p* < 0.001.

**FIGURE 7:**
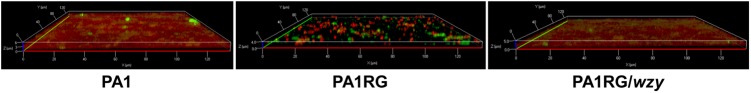
CLSM analysis of biofilm formation. Bacterial cells in biofilm were labeled with the red fluorescent nucleic acid stain SYTO 61, and extracellular polysaccharides were labeled with the green fluorescent stain FITC-ConA. Mutant PA1RG produced thinner biofilm compared to PA1 and PA1RG/*wzy*.

As described in various phage–host interactions, phage predation could pose multifaceted effects on bacterial biology, such as growth, motility, and virulence (Hosseinidoust et al., 2013b; Feiner et al., 2015; De Smet et al., 2017). To assess the potential effects of phage resistance on PA1RG biology, we firstly profiled the growth characteristics of PA1 and PA1RG. The growth rate was not significantly different for the two strains under the tested conditions (**Figure 6A**). However, the ability of mutant PA1RG to produce biofilm was significantly decreased compared with that of the parental strain PA1, and this deficiency could be offset by providing the wild-type gene *wzy in trans* (**Figure 6B**). CLSM analysis of biofilm formation further demonstrated that mutant PA1RG produced less and thinner biofilm compared to both strain PA1 and PA1RG/*wzy* (**Figure 7**), indicating a trade-off between phage resistance and biofilm-forming ability.

## Discussion

Phages are the most diverse and abundant genetic entities on earth with an estimated number of 10^31^ in total (Sharma et al., 2017). The highly broad distribution of phages, particularly for lytic phages, poses a major threat to the survival of their bacterial hosts (Samson et al., 2013). As a result, bacteria have evolved various defense systems to evade and survival phage predation. Known mechanisms include adsorption inhibition, R-M system, CRISPR/Cas system, and Abi system, which function and target every possible step of phage infection (Labrie et al., 2010). Attachment of phage to host cell surface, the initial and essential step to complete phage infection cycle, is usually impaired among phage-resistant bacteria (Labrie et al., 2010). In this study, we elucidated the molecular basis for *P. aeruginosa* PA1RG resistance to phage PaP1. The transition mutation within gene *wzy* resulted in the premature formation of *O*-antigen polymerase, which is involved in long-chain *O*-antigen biosynthesis related to PaP1 recognition and binding. Combined with the previously described resistance mechanism for Red mutant PA1r, which lost a large DNA segment (219.6 kb) containing the key gene *galU* responsible for core oligosaccharide biosynthesis (Le et al., 2014), these results enhanced our understanding of the multiple mechanisms that *P. aeruginosa* used to resist phage predation.

The receptor on the bacterial cell surface is responsible for phage attachment and mirrors the diversity of phage types (Rakhuba et al., 2010). To date, various receptors recognized by *P. aeruginosa* phages have been identified. These receptors include type IV pili (Kim et al., 2012), *O*-antigen (Le et al., 2014), common polysaccharide antigen (Rivera et al., 1992), core oligosaccharide (Temple et al., 1986), and outer membrane protein (Chan et al., 2016b). Interestingly, phages PaP1, K8, and K5, all belonging to the genus of *Pakpunaviruses* and using the *O*-antigen as receptor, infect *P. aeruginosa* strain PA1 and PAK, respectively (Lu et al., 2013; Li et al., 2016c; Pan et al., 2016). Phage-resistant variants possessing mutation in gene *wzy* were selected from both PA1 and PAK upon corresponding phage predation (Li et al., 2016c; Pan et al., 2016), suggesting that *wzy* mutation may represent a preferred resistance mechanism for phages of *Pakpunaviruses*.

The phenotype of phage-resistant Green mutant PA1RG producing decreased biofilm, and the previously identified Red mutant PA1r displaying a growth defect, illustrated the diverse and distinct effects of phage infection on bacteria biology. The similar trade-off between phage resistance and bacteria functionality has been described in various phage–*P. aeruginosa* interactions, which mainly influenced *P. aeruginosa* biofilm formation, virulence, and/or antibiotic resistance (De Smet et al., 2017). The type IV pili dependent phage D3112 selected for *P. aeruginosa* mutants that showed decreased surface adherence and biofilm formation, but increased antibiotic sensitivity (Hosseinidoust et al., 2013a). The use of LPS as receptor by phage E79 resulted in LPS alterations of phage-resistant variants, which in turn lead to decreased swarming and increased biofilm formation (Hosseinidoust et al., 2013a). Serotype conversion of *P. aeruginosa* PAO1 from O5 to O16 as mediated by temperate phage D3 enables bacterial resistance to superinfection by related phages, and also contributes to enhanced adherence and resistance to animal immune systems (Newton et al., 2001; Taylor et al., 2013). Reversion of antibiotic susceptibility was conferred by lytic phage OMKO1 (Chan et al., 2016b), which recognizes and binds to the outer membrane porin M involved in multidrug efflux. OMKO1-resistant mutant exhibited reduced antibiotic resistance owing to the decreased expression of porin M (Chan et al., 2016b). Despite the high conservation between phage PaP1 and K8, mutants against each infecting phage displayed reduced and increased biofilm, respectively (Pan et al., 2016), indicating the conflicting effects of phage predation. Based on the known cases, the diverse effects (either positive or negative) posed by *P. aeruginosa* phages exhibit features of complexity and unpredictability and might be phage and/or host-specific (Chaturongakul and Ounjai, 2014; De Smet et al., 2017).

As natural enemies for bacteria, phages were immediately used to treat bacterial infections upon their discovery approximately 100 years ago (Cisek et al., 2017), and interests in their potential as antimicrobials increasing mainly due to the global threat of antibiotic resistance (Nobrega et al., 2015). To efficiently facilitate the application of phage therapy, a comprehensive understanding of phage–host interactions is required. In this study, we revealed that PaP1 infection resulted in mutant PA1RG with decreased biofilm formation. Biofilm represents an important characteristic that greatly benefits *P. aeruginosa* with defense against antibiotic treatment and prolonged survival especially during chronic lung infection (Stover et al., 2000). The trade-off between phage-resistance and fitness cost may be a potential advantage for phage therapy to treat bacterial infections. Furthermore, a dual therapy with combined phage and antibiotic has displayed increased antibacterial effect compared with the sole use of each agent in several studies (Viertel et al., 2014). For example, methicillin-resistant *Staphylococcus aureus* was efficiently eliminated from diabetic foot infections using phage MR-10 and linezolid (Chhibber et al., 2013), the eradication of *Klebsiella pneumonia* biofilm was increased by the combination of ciprofloxacin and phage KPO1K2 producing the depolymerase (Verma et al., 2010), and limited evolution of bacterial resistance to kanamycin was conferred by phage SBW25Φ2 predation in *P. fluorescens* (Zhang and Buckling, 2012). With the impressive advantages of synergistic antibacterial effect and less frequently evolved resistance (Chan et al., 2016a), the application of phage and antibiotic in combination represents a promising strategy to control bacterial infections, especially for those caused by biofilm-forming and multi-drug resistant bacteria (Tillotson and Theriault, 2013; Parasion et al., 2014).

## Author Contributions

GL, MS, YY, and SL performed the experiments. GL, ML, and SL analyzed the data. SL, JW, YZ, and YT contributed reagents and materials. FH, SL, and GL designed the experiments and wrote the paper.

## Conflict of Interest Statement

The authors declare that the research was conducted in the absence of any commercial or financial relationships that could be construed as a potential conflict of interest. The reviewer KD-W and handling Editor declared their shared affiliation.

## Footnotes

1 http://www.ncbi.nlm.nih.gov/BLAST
